# Stages of development of traditional simulation unit of surgery for manual extra capsular cataract extraction in sub-Saharan African region

**DOI:** 10.11604/pamj.2017.27.261.10980

**Published:** 2017-08-08

**Authors:** Yannick Bilong, Vikas Mahatme, Ngoune Nanfack, Sapate Santosh, Lucienne Assumpta Bella, Côme Ebana Mvogo

**Affiliations:** 1 Department of Ophthalmology, Faculty of Medicine and Biomedical Sciences, University of Yaoundé I, Yaoundé, Cameroon; 2 Founder Medical Director, Mahatme Eye Bank Eye Hospital, Recognised Institute for Post-Graduation, Nagpur, India; 3 Ophthalmology unit, Pediatric and Gynéco-Obstetric Hospital, Yaounde, Cameroon; 4 Surgery simulation unit, Mahatme Eye Bank Eye Hospital, Recognised Institute for Post-Graduation, Nagpur, India

**Keywords:** Surgical simulation, cataract, sub-Saharan Africa

## Abstract

The manual extracapsular extraction of the lens is the surgical technique that is most practiced for the treatment of cataract in sub-Saharan Africa. Learning this technique requires the creation of a surgical simulation unit within training institutes. We describe the development stages of a traditional simulation unit. For this purpose, we present a description of four steps involved in the development of a simulation unit for cataract surgery: the physical creation of the room, the aseptic and antisepsis conditions, the management of the eyes, the development of a curriculum and the administrative policies.

## Introduction

About 50% of blindness in sub-Saharan Africa is caused by cataract [1, 2]; therefore, the extracapsular cataract extraction (ECCE) is a major surgical technique and it is part of the training of an ophthalmology resident. Its apprenticeship ideally begins with the use of surgical simulation sessions [3, 4]. Firstly, these sessions allow appropriation of the rules and gestures of microsurgery in a trusted environment where there is no fear of error. Secondly, they contribute to reducing operative complications for a novice surgeon. This learning ECCE simulation comes in two models with similar results: the model called “traditional” uses physical tools such as artificial eyes, eyes from dead humans or dead animals, whereas the model called “virtual reality” uses a computer simulation program [5]. Not only is the “virtual reality” model very expensive (about 200,000 Euros for installation), it is also designed to be the most expensive with a less accessible surgical technique namely phacoemulsification ECCE [6]. Therefore, it is fair to think that in sub-Saharan Africa, learning the ECCE should be done using the “traditional” way with an emphasis on a manual ECCE instead of phacoemulsification. Hence the interest of this work is to describe our view on the necessary steps for the implementation of a traditional simulation unit (manual ECCE) within a training unit in cataract surgery in sub-Saharan Africa. Our view is a mix of experiences between the **Department of Ophthalmology** of the University of Yaoundé I in Cameroon, and the surgery simulation department designed by Dr. Vikas Mahatme, the Founder Medical Director of Mahatme Eye Bank Eye Hospital and a post graduate teaching institute at Nagpur, India. Indeed, this Indian’s institute practices in a poor socioeconomic context similar to that of sub - Saharan Africa, not only has a strong and long experience in training cataract surgeons, but also has equipped its simulation unit with an economic concern and quality standards in academic and hospital hygiene, which it would be wise to learn from.

## Surgery simulation room

**Figure 1:**
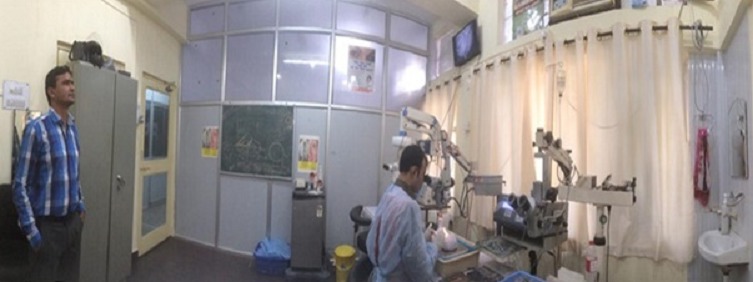
Panoramic view of the simulation room; here, the faculty can watch the intraocular gestures of a learner on a screen (Courtesy of post-doctoral Mahatme Hospital Eye Bank Institute)

**Figure 2:**
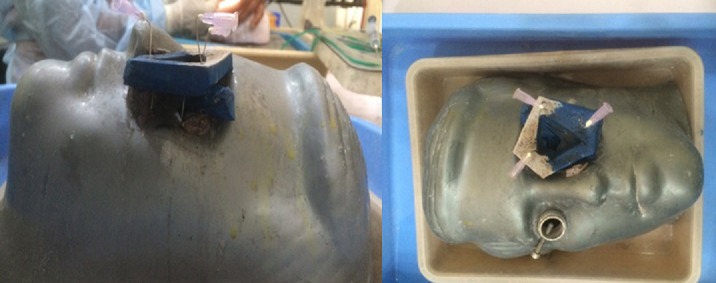
Top view and front view of an example of an attachment with a suitable plastic on a dummy (Courtesy of post-doctoral Mahatme Hospital Eye Bank Institute)

The simulation room (Figure 1) should be spacious so that the movements are comfortable. Firstly, it must contain a blackboard where you can write using different colored chalk. This will allow the instructor to recall the theoretical principles of cataract surgery and also help learners to schematize their questions and difficulties. Secondly, it must contain at least one compound surgery simulation station: an operating microscope connected or not to a TV, a system to stabilize the eye (Figure 2), a full box of cataract surgery, syringes with cannula of 20G, a spray bottle containing water (to hydrate the eye in use), an uncolored gel for the embodiment of the electro-cardiogram (acting as a viscoelastic substance). Thirdly, the simulation room must have instruments wardrobe, a fridge to keep the eyes of animals, garbage cans and a common water source (tap and drainage system of waste water).

## Aseptic conditions and antisepsis

They should approach those of the operating room, due to the manipulation of organic matter, source of infection. Povidone iodine 0.5% or 1% will be used for antiseptic purposes because it does not degrade the corneal epithelial cells and does not induce corneal edema [7]. A resident must already be immersed in the world of hygiene of an operating theater. For this purpose, we think that these following conditions are necessary: The simulation room must be separated from the operating room or any consultation areas and should be well ventilated with a positive air pressure; it is forbidden to eat in the simulation room; the materials cannot be used to perform surgery on humans and must stay in the simulation room ; the eyes of animals and nothing else must be stored in the fridge (the temperature should not induce freezing eyes); the used eyes be collected separately in a specific bin and destroyed by incineration or burial; the room must be sterilized according to its attendance by the steam from the boiling formalin set (For daily use, for example, we advise to do so once a week); at the beginning and end of a working day, we should clean the simulation room with water and antiseptic substance; before each session performed on a given station, the learner must wear a disposable gown and non-sterile gloves; at the end of each session, the learner must wash their hands and materials; and it is forbidden to go to the operating room for surgery on humans, if one has not had a bath and changed his clothes.

## Management of the eye

In our socio-economic and cultural context we can collect eyes from sheep, cows, rabbits and pigs. These eyes will be obtained from the nearest butcher, with an integrated sclerocorneal wall, the bulbar conjunctiva in place, oculomotor insertion tendons of the muscles and optic nerve severed. They must be used within the first 48 hours following the slaughter of the animal. Their conservation will be made fresh in a refrigerator. Tap water is used for hydration of the eye and the reform of the anterior chamber because it is inexpensive and induces a mild corneal thickening while preserving intact the crystalline lens cells [8]. If stored beyond 48 hours, with our experience, the practical steps of the surgery will be difficult because on one hand the sclera and zonule become very fragile and on the other hand because of the increased risk of infection in the handling of decaying organic matter.

Several methods exist for eye stabilization [9], but the use of a dummy head is by far the most suitable to our context. It is easy for oneself to design one, using clay or to adapt a plastic from the exhibition dummies used in clothes shops. This stabilization may be accomplished by fixing animal eye on a ring-shaped carrier (example: cut sole of a sports shoe worn). For stabilization, use needles up operations on tendon sheaths of the rectus muscles (Figure 2). The main disadvantage of this method is the possible instability of the globe during handling and loss of tone of the globe requiring intravitreous injections of water or viscoelastic substance [9].

The animal eye used for learning by simulation of the ECCE is anatomically different from an adult human eye that has a cataract. Firstly, the first dimensions of the cornea and the depth of the anterior chamber are different. Secondly, the eye of the animal used is often devoid of cataract and has an elastic and thick anterior capsule [9]. Due to these different aspects during the simulation sessions, we observed that: handling instruments in the anterior chamber in animals and humans is not strictly the same; the extent of acts (incisions outside and inside the eye) is greater on the animal eye relative to the human eye; handling an elastic anterior capsule of the animal gives rather experience for a cataract surgery in children.

That last observation have encouraged the development of various methods for inducing in an animal eye, through precipitation and cross-linking of proteins, cataract and make less elastic the anterior capsule [9]. But in practice, according to our experience, where the resident has access directly to a human eye whenever he has mastered the depth perception of spaces under the microscope and implementing principles of such a move described by the curriculum, the use of such methods is useless. In fact, in the operating room under supervision, he will adapt quickly and confidently to the human eye with minimal chance of inducing irreversible per operative complications.

## Learning curriculum and administration policies

The resident must always have a curriculum serving as a guide and the criteria of the step by step assessment of his requires surgical skills are those recommended by the International Council of Ophthalmology (ICO) [10], for example. According to our experience the most important in a curriculum is regularly repeating certain gestures within a given period of time till they are mastered in the simulation room as defined by the ICO. Then, step by step the learner will perform surgery on a patient under supervision while at the same time overseeing his less experienced classmates during simulation sessions in order to preserve the skills that they have already acquired.

It is important to define administrative management of the learning room simulation, to ensure the sustainability of its operation. We observed that, it is difficult in practice to an ophthalmic surgeon to give of his time to be with residents in the simulation room. Indeed, he will rather prefer to treat patients and supervise students when they are eligible to transition to the operating room. Therefore, we think, it is convenient for a training institute to choose one of two following options: Train a non-ophthalmologist staff (e.g. an ophthalmic nurse) in cataract surgery and permanently assign them to supervise residents in the simulation room. Or, define a period of time during each academic year where an ophthalmology faculty will focus in training a first wave of senior residents, who in turn will train their juniors on a daily basis. The ophthalmology faculty will have to evaluate the overall progress of all the residents from time to time and make the necessary adjustments to address noticeable gaps.

In addition, it is important for us to obtain sources of financing for the acquisition of major equipment (operating microscope, video surveillance, stabilization apparatus of the eye, etc.) and consumables (eyes of animals, surgical instruments, suture son, needles, syringes, gloves, etc.). Sometimes, in the absence of funding, we seek for donations of worn or amortized consumables and equipment from large surrounding hospitals performing eye surgery.

## Conclusion

The development of a learning manual simulation unit of ECCE is essential in the teaching of cataract surgery. This unit must respect certain principles of universal creation and managements, which are feasible in our poor socio-economic context of sub -Saharan Africa.

## Competing interests

The authors declare no competing interest.
